# Does Low Grade Systemic Inflammation Have a Role in Chronic Pain?

**DOI:** 10.3389/fnmol.2021.785214

**Published:** 2021-11-10

**Authors:** Wen Bo Sam Zhou, JingWen Meng, Ji Zhang

**Affiliations:** ^1^The Alan Edwards Centre for Research on Pain, McGill University, Montreal, QC, Canada; ^2^Faculty of Dentistry, McGill University, Montreal, QC, Canada; ^3^Department of Neurology and Neurosurgery, Faculty of Medicine McGill University, Montreal, QC, Canada

**Keywords:** systemic chronic inflammation, chronic pain, psychological disorders, systemic modulation, peripheral neuropathy, cytokines, chemokines

## Abstract

One of the major clinical manifestations of peripheral neuropathy, either resulting from trauma or diseases, is chronic pain. While it significantly impacts patients’ quality of life, the underlying mechanisms remain elusive, and treatment is not satisfactory. Systemic chronic inflammation (SCI) that we are referring to in this perspective is a state of low-grade, persistent, non-infective inflammation, being found in many physiological and pathological conditions. Distinct from acute inflammation, which is a protective process fighting against intruders, SCI might have harmful effects. It has been associated with many chronic non-communicable diseases. We hypothesize that SCI could be a predisposing and/or precipitating factor in the development of chronic pain, as well as associated comorbidities. We reviewed evidence from human clinical studies indicating the coexistence of SCI with various types of chronic pain. We also collated existing data about the sources of SCI and who could have it, showing that those individuals or patients having SCI usually have higher prevalence of chronic pain and psychological comorbidities. We thus elaborate on the need for further research in the connection between SCI and chronic pain. Several hypotheses have been proposed to explain these complex interactions.

In response to infection or injury, the body reacts quickly with an activation of immune cells - mostly myeloid cells, an increase in the production and release of inflammatory mediators, and an increase of phagocytic activity to remove foreign bodies or tissue debris to promote healing. Acute inflammation following exposure to pathogen-associated molecular patterns (PAMPs) and damage-associated molecular patterns (DAMPs) is a physiological process that the body uses to fight against intruders and pathogens. It however needs to be tightly regulated to prevent undesirable, maladaptive consequences. Failure in resolving inflammation and/or long-term, repeated stimulation of the immune system may lead to **systemic chronic inflammation (SCI)**, sometimes also called subclinical inflammation. Contrary to acute inflammatory reaction, SCI is characterized by its long-lasting and low-grade profile. Despite many canonical biomarkers for acute inflammation being not or barely detectable in SCI (Furman et al., 2019), this chronic inflammation contributes to the disruption of a finely tuned interconnected physiological network, through its collateral effects on multiple systems/organs (Sturmberg et al., 2017). Certain social, environmental factors and lifestyle changes have been found to influence the establishment of SCI. Several chronic diseases including cardiovascular diseases, cancer, depression, metabolic and neurodegenerative disorders have been closely associated with SCI (Libby, 2006; Kressel et al., 2009; Grivennikov et al., 2010; Ferrucci and Fabbri, 2018; Walker et al., 2019). While acute inflammation is a protective mechanism, SCI appears to be health damaging, having significant impact on the rise of many non-communicable physical and mental problems that dominate global rates of morbidity and mortality in the modern society (Bennett et al., 2018). It becomes pressing imperative to explore and understand the involvement of SCI in health and diseases.

**Chronic pain** is a debilitating disorder, accounting for around one fifth of physician visits across the world (Treede et al., 2015). Recent population-based estimates of chronic pain among U.S. adults are about 20%, with higher prevalence among women and older adults (Dahlhamer et al., 2018; Pitcher et al., 2019). It has an enormous economic burden on the society due to direct medical costs and loss of productivity. Despite extensive efforts in global research to advance our understanding of the underlying mechanisms, current pain management is far away from satisfaction, because of either lack of efficacy or severe side effects. Chronic pain remains a global public health problem with vast unanswered questions and unmet needs (Yekkirala et al., 2017). Neuropathic pain is the most difficult chronic pain condition to manage where peripheral neuropathy is the main culprits. A variety medical conditions could cause peripheral neuropathy, either mononeuropathy following trauma or even just surgery, or polyneuropathy which could be associated with diabetes, chemotherapy, alcohol abuse or autoimmune conditions. However not all peripheral neuropathy patients, either from trauma, surgery, or diabetes develop chronic neuropathic pain. Acute pain after surgery or injury is almost ubiquitous, yet of 320 million people having surgery each year, more than 10% of these patients develop chronic, essentially neuropathic post-surgical pain (Kehlet et al., 2006; Macrae, 2008; Borsook et al., 2013). While peripheral neuropathy is a common complication of diabetes, which affects over 90% of the diabetic patients (Tesfaye et al., 2013), 10–26% of this population suffer from chronic pain (Low and Dotson, 1998). It is thus necessary to decipher the risk factors predisposing the transition from acute pain to chronic neuropathic pain and understand the underlying mechanisms of painful and non-painful peripheral neuropathy.

Other than peripheral neuropathy-triggered neuropathic pain, chronic widespread pain (CWP), including fibromyalgia (FM), is another chronic pain condition where the etiology is unknown. Characterized by long-lasting, diffused pain in the body, it is frequently associated with fatigue and psychological distress, significantly affecting patients’ quality of life. With high prevalence rates of 10% for CWP (Croft et al., 1993; Mourão et al., 2010) and 2% for FM, mainly in women (Häuser et al., 2014), this type of “inexplicable” chronic pain presents a major, untreatable health burden that poses a serious challenge for clinicians. While recent investigation established a central sensitization feature in CWP (Meeus and Nijs, 2007; Yunus, 2007; Turk et al., 2016; Ji et al., 2018), it remains to identify the potential peripheral contribution, since ongoing peripheral input seems important for the maintenance of central hyperexcitability (Staud et al., 2009).

Furthermore, not all chronic pain is the same. Beyond physical pain, some chronic pain patients often suffer from psychological symptoms, such as depression and anxiety; while others have reported that their chronic pain severely interfere with their life and work activities. The latter has been recognized recently as “high-impact chronic pain” that accounts for 8% of U.S. adults (Dahlhamer et al., 2018). Chronic pain has been found more frequently associated functional disability than those with stroke or kidney failure (Pitcher et al., 2019). Psychological distress has also been suggested as an important link between physical pain and dysfunction (Von Korff and Simon, 1996; Jensen et al., 2011; Turk et al., 2016; Gandolfi et al., 2021). The risk factors for individuals that are prone to developing psychological and functional comorbidities remains to be identified.

Notwithstanding the recognition of the increasing importance of the SCI in chronic diseases over past two decades, the potential contribution of SCI in chronic pain and associated dysfunction have still been largely overlooked. While several risk factors for developing chronic pain, such as genetic traits and patients’ psychological status, have been suggested and investigated previously (Glare et al., 2019), we hypothesize that SCI could be a predisposing and/or precipitating factor in the development of chronic pain. Our primary goal is to increase the awareness on the potential contribution of SCI in chronic pain. In this perspective, we will first describe evidence from human clinical studies suggesting the coexistence of SCI with various types of chronic pain; secondly, we will collate existing data about the sources of SCI and who could have it; we will then discuss knowledge gap and future directions to better understand how SCI influence chronic pain and associated comorbidities.

## Do Chronic Pain Patients Have Systemic Chronic Inflammation?

SCI has been reported in various chronic pain patients. Strikingly, this is not only observed in painful conditions associated with inflammatory pathology, such as rheumatoid arthritis (Burmester et al., 1997; Stuhlmüller et al., 2000; McInnes, 2001; Alivernini et al., 2020) and chronic inflammatory demyelinating polyneuropathy (CIDP) (Svahn et al., 2014; Beppu et al., 2015). In several clinical studies, by analyzing mRNA and protein levels of inflammatory cytokines in patients’ blood samples, Sommer, Üçeyler and their colleagues have continually provided direct evidence that a pro-inflammatory profile is associated with painful peripheral neuropathy. The mRNA and/or proteins of proinflammatory cytokines IL-2, TNF-α and IL-1β are higher in painful peripheral neuropathy patients than those in painless neuropathy patients and healthy controls (Uçeyler et al., 2007b; Held et al., 2019), while those of anti-inflammatory cytokines IL-10 and IL-4 are similar or lower in painless neuropathy patients and healthy controls (Uçeyler et al., 2007b). More pro-inflammatory cytokines are elevated in peripheral blood mononuclear cells (PBMC) of patients with painful polyneuropathy (PNP) than in patients with painless PNP when compare with controls (Langjahr et al., 2018). They also found a proinflammatory cytokine feature in patients with complex regional pain syndrome (CRPS) (Uçeyler et al., 2007a). Such systemic inflammation signature has been corroborated by other investigators in patients suffering from diabetic neuropathic pain (Doupis et al., 2009) and pain associated with radiculopathy (Moen et al., 2016). In addition, signs of ongoing systemic inflammation have also been repeatedly detected in CWP patients (Gerdle et al., 2017; Wåhlén et al., 2018). In fibromyalgia patients, levels of pro-inflammatory cytokines are increased while levels of anti-inflammatory cytokines are reduced (Uçeyler et al., 2006; Ernberg et al., 2018). In acute and chronic low back pain patients, although distinct, an unbalanced pro-inflammatory and anti-inflammatory profile was found in both groups (Teodorczyk-Injeyan et al., 2018). Even though causal relationship remains to be determined, it appears that SCI could co-exist with many non-inflammatory chronic pain conditions.

While most the aforementioned clinical studies have limited their investigations in few inflammatory markers, some recent studies detailed the screening with 92 multiplex panel for inflammation related blood proteins consisting of cytokines, chemokines and growth factors. In a group of fibromyalgia patients, at least a dozen inflammation related proteins are elevated in both the CSF and serum of the patients (Bäckryd et al., 2017). In another group of severely impaired chronic pain patients, significant difference was found in 43/92 inflammatory biomarkers. Three of these markers (CXCL5, SIRT2, and AIXN1) were eight times higher than the controls, confirming that these chronic pain patients suffer from low grade SCI (Hysing et al., 2019). Even more remarkable, this study followed up with patients after one-year-participation in multimodal pain rehabilitation program. They reported that among patients whose pain, fatigue and cognitive impairment improve, most of their inflammation related proteins tend to be normalized (Hysing et al., 2019). In a prospective study with a mean of 6.5-year follow-up in an older general population, Herder et al. (2017) demonstrated that low grade SCI precedes both the onset and the progression of distal polyneuropathy. Higher IL-6 and TNF-α levels are associated with the incidence of developing neuropathy, while higher systemic levels of sICAMP-1 and IL-1RA are associated with the disease progression. They suggest that SCI could predict the onset and progression of such pathology, including diabetic neuropathy. Another 6-year longitudinal study on elders (average age: 69.05 years), predominantly women (71.5%) under chronic stress, shed light on the relationship of pain, hostility and SCI (Graham et al., 2006). They demonstrated that greater perceived pain, to a lesser extent, hostility, were associated with an increase of blood CRP levels. By using a multivariate bi-directional model, their results further support that SCI may act as functional link between pain and hostility, where SCI could increase pain directly or indirectly via psychological responses, significantly impacting patients’ quality of life and overall health. Furthermore, in a randomized and controlled study, Lasselin et al. (2016) reported that low-grade systemic inflammation could abate the effect of behavioral treatment for chronic pain in adults. They found that higher levels of baseline TNF-α and IL-6 in the blood of patients before behavioral intervention were associated with less improvement in pain intensity and psychological inflexibility; and pre-treatment inflammatory scores were negatively associated with changes in mental health-related quality of life. These types of prospective studies, especially with intervention, are very promising for pinpointing the potential involvement of SCI in chronic pain.

The theoretical link between systemic inflammation and pain response has also been explored in human experimental studies. Intravenous injection of lipopolysaccharide (LPS) (0.8ng) in healthy individuals increased widespread musculoskeletal pain sensitivity, which was correlated with changes of circulating IL-6 (Wegner et al., 2014). LPS (0.6 ng, i.v.) not only induced experimental systemic inflammation, increased pain sensitivity in healthy subjects, but also impaired their brain neuronal circuits involved in pain signal transmission and processing (Karshikoff et al., 2016). In a population-based study of inflammation and pain sensitivity, a relationship between systemic inflammation and pain tolerance/thresholds has been demonstrated in a large cohort (*n* = 827) of healthy adolescents (15–19 years). Among 119 inflammatory serum biomarkers, all fatty acids and 10 proteins are anti-inflammatory and protective where higher levels of these proteins are associated with increased cold-pressor pain tolerance, and two are associated with lower tolerance. A similar correlation has been found for heat and pressure pain tolerance and thresholds. These data indicate that in young healthy individuals, anti-inflammatory profile predominates in the circulation, and of paramount importance, it is analgesic (Iordanova Schistad et al., 2020).

## Where Does the Systemic Chronic Inflammation Come From and Who Could Have It?

While an increasing body of evidence suggests the presence of SCI in chronic pain patients, these data also lead to more questions such as the sources of SCI and which individuals in the general population are at higher risk of having it. A long list of potential endogenous and exogenous factors contributing to the establishment of SCI have been identified (Furman et al., 2019). These could include, aging-related cell senescence, un-resolved or chronic infection, unhealthy lifestyle habits, microbiome dysbiosis, challenges on social and cultural status, environment pollutants, etc. Here we will focus more specifically on several conditions associated with high prevalence of chronic pain. It is, however, worthwhile to recognize that although most of SCI evidence have been collected from blood samples, and the blood has been used as window for global immune system analysis in humans. The SCI that we discuss here is not restricted only to immune cells in the blood, but rather it is a multifactorial systemic process, involving the production of pro- and anti-inflammatory mediators from different organs and tissues, and their complex interactions through cell-to-cell crosstalk.

SCI in the aging population has been well established. We age along with inflammatory signatures. Coined as “inflammaging” since 2000 (Franceschi et al., 2000), it has been thoroughly investigated in the last two decades. During the course of aging, this low-grade chronic inflammation is built up by the repeated and persistent stimulation of endogenous stimuli related to cell senescence, likely assisted by some exogenous factors, such as chronic stress, unhealthy life style or environmental changes (Zhu et al., 2014). Various changes in immune cell phenotype and function, as well as the increase of many major proinflammatory molecules, mainly but not limited to cytokines such as IL-6, TNF-α, IL-α and IL-18, has been found in healthy elderly individuals (Roubenoff et al., 1998; Morrisette-Thomas et al., 2014; Ferrucci and Fabbri, 2018). They inevitably play roles in many age-related diseases (Rea et al., 2018). The fact that concomitant and abundant increases of both pro- and anti-inflammatory molecules in the blood of centenarians (Franceschi et al., 2007; Santos-Lozano et al., 2020), who have been largely exempted or delayed chronic pathologies, further suggests that inflammaging is not just about having higher levels of proinflammatory mediators in the blood. Indeed, what matters the most for a healthy and successful aging is the balance between pro- and anti-inflammatory responses (Franceschi et al., 2007; Morrisette-Thomas et al., 2014). Aging population usually suffers from more chronic pain. The worldwide prevalence of chronic pain is estimated to be between 25 and 50% in elderly people living in the community and up to 83% in those living in nursing homes (Won et al., 2004; Zanocchi et al., 2008; Cravello et al., 2019). This is sharply contrasted to that in young adults, which typically lies between 7 and 22% (Moulin et al., 2002; Bouhassira et al., 2008; Schopflocher et al., 2011; Von Korff et al., 2016; Dahlhamer et al., 2018). The high prevalence of chronic pain in aging population reaches a plateau at around 70–75 years (Gibson and Schroder, 2001). Elders report greater levels of pain intensity as compared to younger ones (Nahin, 2015). Pain is one of the most widely cited symptoms underlying disability among older adults (Patel et al., 2013), impacting their quality of life (Lee H. S. et al., 2018). With aging, chronic pain experience is associated with higher risk for depression (Turk et al., 1995; McCarthy et al., 2009; Bauer et al., 2016), accelerating decline in physical function and increase in the number of comorbid symptoms (Okabe et al., 2017), including precipitated memory decline and increased probability of dementia (Whitlock et al., 2017). While increasing evidence strongly implies SCI as a driver to most chronic diseases of older age and as the root cause of decreased cellular responsiveness (Furman et al., 2019), it remains elusive on its role in the development and progression of chronic pain as well as psychosocial comorbidities in elders.

Metaflammation is a term defined as a state of chronic metabolic inflammation, evoked often by the consumption of calorically rich diets leading to chronic overnutrition, combined with a sedentary lifestyle typical in Western societies (Gregor and Hotamisligil, 2011). Qualitatively and quantitatively, several components in Western diets, such as an abundance of fats, sugars and salts, are pro-inflammatory (Christ et al., 2019; Fan et al., 2020). These molecules could disturb the gut barrier integrity and contribute to dysbiosis (David et al., 2014). Recent evidence also points out that such impacts may not necessary be transient but can have a lasting effect through “trained immunity”, where innate immune cells have been reprogrammed, likely epigenetically, and will become more easily activated even after the systemic inflammation is gone (Christ et al., 2018; Netea et al., 2020). These epigenetic alterations are even heritable after multiple generations, predisposing future generations to have systemic inflammation or easily activated inflammatory responses (Claycombe et al., 2015; Tiffon, 2018; Greco et al., 2019). Furthermore, obesity is one of the major metabolic disorders. It has been accepted for decades that obese people live with a pro-inflammatory tone. The levels of some inflammatory molecules such as cytokines, chemokines, C-reactive protein (CRP), Serum amyloid A (SAA) and fibrinogen were found higher in the serum of obese individuals than non-obese counterparts (Michalovich et al., 2019). Interestingly, a 10% weight loss reduced plasma concentrations of several cytokines in obese women (Marfella et al., 2004). Low-grade SCI has also been extensively linked to disturbances in glucometabolic pathways as observed in people with type 2 diabetes (T2D), with the most consistent being for leukocytes and the strongest for CRP (Duncan et al., 2003; Noordam et al., 2018; Elimam et al., 2019; Scheithauer et al., 2020). In addition, proinflammatory cytokines IL-6, IL-8 and TNF-α, chemokines CCL1, CCL2, CCL4, CCL5, CCL11, CXCL8, CXCL10, and CX3CL1, and even Granzyme B, a serine protease secreted by lymphocytes and macrophages, were found elevated in the serum of T2D patients (Cimini et al., 2019; Pan et al., 2021). However, a protective association of anti-inflammation with T2D was also revealed, since adiponectin, an adipocyte-secreted, anti-inflammatory protein was found indeed lower than that in healthy controls (Duncan et al., 2003). It has been reported from a survey of over 1,000,000 individuals in the US, that BMI and pain perception is strongly correlated. People who are obese are considerably more prone to having daily pain, and such association rises with age (Stone and Broderick, 2012). Obesity has often been tied with low back pain (Hashem et al., 2018), chronic musculoskeletal pain (Cooper et al., 2018), fibromyalgia (Correa-Rodríguez et al., 2019), and osteoarthritis (Koonce and Bravman, 2013). Thus, obesity is now considered an important pain facilitator (Eichwald and Talbot, 2020). While there is an ever-increasing prevalence of T2D in modern society, diabetic neuropathy is one of the major reasons for which patients to seek physicians’ help. Although many patients with diabetic neuropathy may be entirely asymptomatic, approximately 15–25% of T2D patients present with neuropathic pain (Harris et al., 1993; Davies et al., 2006; Sadosky et al., 2008). It is still not clear why some patients develop neuropathic pain, while others with a similar degree of neuropathy do not. Thus, it remains to be clarified whether and to what extent the established SCI could facilitate the development of painful neuropathy. Proper regulation on the interaction between the immune system and the body metabolism is crucial for health and has important implications for many pathological conditions, including metabolic disorder-associated neuropathic pain.

It appears that individuals living with chronic psychological distress usually has a pro-inflammatory background. The first series of reports dated in 1990s showed that some psychiatric disorders are accompanied with activation of inflammatory pathways. Proinflammatory cytokines were found elevated in the blood samples of bipolar disorder, schizophrenia, and post-traumatic stress disorder (PTSD) patients (Spivak et al., 1997; Rapaport and Bresee, 2010; Kirkpatrick and Miller, 2013; Michopoulos et al., 2015, 2017; Ascoli et al., 2016; Chen P. et al., 2020). For example, a meta-analysis of 20 studies investigating plasma and serum found PTSD to be associated with elevated levels of circulating peripheral IL-6, IL-1β, TNFα, and IFN-γ (Passos et al., 2015). Since then, increasing evidence has also revealed that depression and anxiety are tightly associated with low grade SCI (Maes et al., 1995; Dowlati et al., 2010; Berk et al., 2013; Mazza et al., 2020; Poletti et al., 2021). Although recent studies reported that depression could be predisposed by childhood SCI (Dutcher et al., 2020; Cao et al., 2021), it is still equivocal whether SCI is depressogenic and anxieogenic. Intriguingly, antidepressants, especially selective serotonin reuptake inhibitors (Tynan et al., 2012; Gałecki et al., 2018; Hou et al., 2019; Wang et al., 2019; Dionisie et al., 2021) and TCA (Xia et al., 1996; Maes et al., 1999; Kenis and Maes, 2002; Baumeister et al., 2016; Faissner et al., 2017), have powerful anti-inflammatory properties by decreasing the production/release of proinflammatory mediators and simultaneously increasing that of anti-inflammatory cytokines. Moreover, physical activities have been found to significantly reduce SCI and people engaging in regular activities are less likely to develop future depressive symptoms (Hamer et al., 2009; Frank et al., 2019). Psychological stress, either acute or chronic, can stimulate systemic inflammation. Chronic pain and psychological impairment are usually bidirectionally comorbid and reciprocally connected (Tegethoff et al., 2015). While depression and anxiety have been listed as predisposing risk factors for chronic pain, they are also the most frequent psychological comorbidities of chronic pain (Kato et al., 2006; Tunks et al., 2008; Davis et al., 2011; Tegethoff et al., 2015; Desai et al., 2020). These comorbid symptoms of anxiety and depression are associated with decreased chances of recovery (Nordstoga et al., 2017; Roughan et al., 2021). It has been reported that psychological interventions such as cognitive behavioral therapy for chronic pain provide small benefits in reducing pain, disability, and distress, or even with worse outcomes if a pro-inflammatory state is present (Lasselin et al., 2016; Williams et al., 2020). It is thus plausible to hypothesize that SCI may act as a functional link between chronic pain and psychosocial distress. Further investigations are needed to better understand whether pre-existing psychological distress-associated SCI promotes acute to chronic pain transition or worsens the prognosis of chronic pain, or verse versa, chronic pain-associated SCI increases and exacerbates psychological comorbidities?

## Perspective: How Do Systemic Signals (Systemic Chronic Inflammation) Impact Chronic Pain?

We discussed above that those individuals or patients having SCI usually have higher prevalence of chronic pain and associated comorbidities. However, the foremost crucial question is whether and how this proinflammatory fingerprint is directly involved and contributing to the pathophysiology of chronic pain. As of now, most of the current clinical evidence is cross-sectional, only provides correlations between SCI and various pain conditions. Some additional prospective and interventional clinical studies are necessary, especially those with large sample sizes and multiple molecular panels, to answer directly to this question. Given that chronic pain has complex multifactorial etiologies and aspects, if possible, intensity, duration of physical pain, psychological comorbidities and functional disability should all be taken into consideration as a whole in clinical investigation. While up to date, very limited preclinical studies or evidence involving SCI and chronic pain are available in the literature, using various well established animal models of chronic pain to investigate the potential contribution of SCI will definitely provide complementary information to human-based studies. It could help to overcome some limitations in human studies, for example restrictions due to ethical reasons or difficulties in the recruitment of patients, etc. Moreover, establishing a SCI status in humans could potentially take months to years due to its chronic nature, but this could be significantly shortened in rodents with a shorter life span (2 years in rats/mice vs. > 70 years in humans). The use of animal models could accelerate our understanding on the process of SCI (from the initiation to the maintenance) and its interaction with a whole, living biological system. Without doubt, animal studies are important and crucial in the exploration of pathological mechanisms to understand how systemic signals from SCI impact pain behavior. Animal studies will have an indispensable role in defining the causal relationship between SCI and different types of chronic pain.

Before determining how systemic signals reach the nervous system, it is essential to decipher cellular and molecular components making up the SCI. Advances in “-omics” technologies provide scientists with the ability to probe the biologic variance in either human or animal samples with high sensitivity at the single-cell level (Sun and Hu, 2016; Yugi et al., 2016; Chappell et al., 2018). These approaches have been of particular benefit to study and characterize the potential of SCI modulation in chronic pain. Deep profiling of peripheral immune cells and peripheral blood with, for example, cutting-edge single-cell transcriptomics, multiplex assays, and plasma or serum proteomic analysis would pave the way to identify the underlying connectivity between the immune system and the nervous system.

There are many different hypotheses as to how SCI could potentially alter or be a contributing factor to chronic pain. In physiological conditions, other than some specific and restricted areas such as nerve terminals and dorsal root ganglia (DRG), most parts of the nervous system along the pain transmission pathway are well protected by blood nerve barrier (BNB), blood spinal cord barrier (BSCB), and blood brain barrier (BBB). Whereas peripheral nerve endings (nociceptors) and the DRG (the somata of nociceptors) are directly exposed to circulating products and other danger signals, it is however unclear whether the repeated, chronic stimulation by the low-grade SCI is sufficient to excite nerve endings or prime the sensory neurons. Furthermore, while some studies revealed that aging (Montagne et al., 2015; Erdõ et al., 2017) and obesity (Salameh et al., 2019; Yamamoto et al., 2019) are associated with neurovascular dysfunction and BBB disruption in certain CNS regions, there is, to the best of our knowledge, no data available in the literature regarding the potential impact of SCI on the integrity of the BNB, BSCB, and BBB, especially in pain related brain areas. Thus, whether low-grade SCI is able to breakdown or loosen the barriers in chronic pain conditions remains to be determined. In the last two decades, it has been well documented and accepted that neurons are not the only cell type involved in chronic pain. Neuroinflammation contributes to the initiation and maintenance of chronic pain (Ji et al., 2016; Sommer et al., 2018). Spinal microglia and astrocyte activation enhances central sensitization by the release of various inflammatory mediators (Zhang and De Koninck, 2006; Echeverry et al., 2017; Ji et al., 2019; Donnelly et al., 2020). Peripheral immune cells recruited and activated in the injured nerve participate in amplifying peripheral sensitization (Morin et al., 2007; Lee S. et al., 2018; Chen O. et al., 2020). While we are expecting direct evidence that systemic signals from SCI can boost neuroinflammation to increase peripheral and/or central excitation, Karshikoff et al. (2015) reported that systemic inflammation modulates brain circuits involved in descending pain inhibition, rendering sick individuals to be more sensitive to painful stimuli. All in all, there remains much to learn and to understand regarding the communication between the immune system via systemic signals in the case of SCI, and the nervous system toward pain circuits for chronic pain. In addition to further collecting clinical data, preclinical studies are largely awaited on this topic. However, to warrant translational value, appropriately mimicking SCI in a proportionate way is crucial to avoid an oversimplified conclusion.

Disentangling the contribution of potentially mutually interacting factors is difficult. As we move forwards in understanding the contribution of SCI to chronic pain, it becomes more and more evident that complex networks form the basis of physiological functions and dysfunctions. Unmasking low-grade SCI as a contributor to chronic pain and associated comorbidities will advance our understanding on the disease pathogenesis. Some key inflammatory pathways may influence the trajectories of these complex and elusive disorders. Due to low-grade and sub-clinical nature of SCI, direct pharmacological targeting might not be the first choice, however, the fact that many chronic pain conditions co-exist with SCI should not be neglected as it might modulate the efficacy of treatments. On the other hand, non-pharmacological interventions, such as promoting a healthy lifestyle, seems promising in reducing SCI. Various studies have shown that healthy lifestyle with exercise (Gleeson et al., 2011; Woods et al., 2012) and consuming certain types of diets (Maleki et al., 2019; Şanlier et al., 2019; Tolkien et al., 2019) are anti-inflammatory and can reduce chronic inflammation. Altogether, a better understanding of SCI and its potentially harmful effects could reveal unknown or overlooked mechanisms of chronic pain and diminishing SCI would contribute to improving the individuals’ quality of life, including reducing chronic pain and its comorbidities.

## Data Availability Statement

The original contributions presented in the study are included in the article/supplementary material, further inquiries can be directed to the corresponding author/s.

## Author Contributions

WZ and JM participated in manuscript drafting. JZ wrote the manuscript. All authors reviewed the manuscript and discussed the work.

## Conflict of Interest

The authors declare that the research was conducted in the absence of any commercial or financial relationships that could be construed as a potential conflict of interest.

## Publisher’s Note

All claims expressed in this article are solely those of the authors and do not necessarily represent those of their affiliated organizations, or those of the publisher, the editors and the reviewers. Any product that may be evaluated in this article, or claim that may be made by its manufacturer, is not guaranteed or endorsed by the publisher.
